# Leukocyte telomere length is associated with iron overload in male adults with hereditary hemochromatosis

**DOI:** 10.1042/BSR20201916

**Published:** 2020-10-23

**Authors:** Maximilino Martín, Andrea Millan, Florencia Ferraro, Walter F. Tetzlaff, Ezequiel Lozano Chiappe, Eliana Botta, Marcelo Castro, Laura Boero, Jorge Rey, Jorge Daruich, Gustavo Frechtel, Tomas Meroño, Gloria Cerrone, Fernando Brites

**Affiliations:** 1 Laboratorio de Lípidos y Aterosclerosis, Facultad de Farmacia y Bioquimica, Universidad de Buenos Aires, Buenos Aires, Argentina; 2 Laboratorio de Genetica, Facultad de Farmacia y Bioquimica, Universidad de Buenos Aires, Buenos Aires, Argentina; 3 Hospital de clínicas ‘José de San Martín’, Departamento de Hemoterapia e Inmunohematología, División de Transfusión y Transmisión de Enfermedades, Universidad de Buenos Aires, Buenos Aires, Argentina

**Keywords:** Iron Metabolism, Iron Overload, Telomere Lenght

## Abstract

**Background:** Hereditary hemochromatosis (HH) is a primary iron overload (IO) condition. Absolute telomere length (ATL) is a marker of cellular aging and DNA damage associated with chronic diseases and mortality.

**Aim:** To evaluate the relationship between ATL and IO in patients with HH.

**Methods:** Cross-sectional study including 25 patients with HH: 8 with IO and 17 without IO (ferritin < 300 ng/ml) and 25 healthy controls. Inclusion criteria were: age > 18 years, male sex and HH diagnosis. Patients with diabetes or other endocrine and autoimmune diseases were excluded. ATL was measured by real-time PCR.

**Results:** HH patients with IO were older (*P*<0.001) and showed higher ferritin concentration (*P*<0.001). Patients with HH, disregarding the iron status, showed higher glucose and body mass index (BMI) than controls (both *P*<0.01). ATL was shorter in patients with IO than controls [with IO: 8 (6–14), without IO: 13 (9–20), and controls: 19 (15–25) kilobase pairs, *P*<0.01]; with a linear trend within groups (*P* for trend <0.01). Differences in ATL remained statistically significant after adjusting by age, BMI and glucose (*P*<0.05).

**Discussion:** Patients with IO featured shorter ATL while patients without IO showed only mild alterations *vs.* controls. Screening for IO is encouraged to prevent iron-associated cellular damage and early telomere attrition.

## Introduction

Hereditary hemochromatosis (HH) is a primary condition, which might lead to iron overload (IO) depending on host and ambient factors. Two main types of HH have been described: (1) the recessive type which involves genes that are related to the activation of hepcidin such as the human homeostatic iron regulator (*HFE*), hemojuvelin (*HJV*), and hepcidin (*HAMP*) genes; and (2) the dominant type which involves the hepcidin receptor ferroportin (*FPN*). Among these, the main gene associated with HH is *HFE*, located in the short arm of chromosome 6 (6p). The HFE gene encodes a protein that is located on the surface of cells, primarily liver, intestinal and immune cells. This protein interacts with other proteins on the cell surface to detect the amount of iron in the body. When the HFE protein is bound to the transferrin (Tf) receptor (TfR) 1, the receptor cannot bind to Tf preventing the entrance of iron to liver cells. Furthermore, *HFE* protein regulates the production of hepcidin. Hepcidin is produced by the liver, and it regulates iron absorption from the diet and its release from storage sites in the body. When the HFE protein is not bound to TfR 1, it binds to a group of other proteins that include hepcidin triggering the production of hepcidin. Therefore, binding of HFE protein to TfR 1 represses hepcidin expression. Consequently, current models of iron metabolism regulation propose that highly iron-saturated Tf would release HFE protein from TfR1 increasing iron uptake by cells and hepcidin up-regulation [1,2].

Even if iron is essential for cells, it may also be involved in the generation of reactive oxygen species leading to cellular damage [3–7]. In patients with IO, the hepatocytes, cardiomyocytes and cells from endocrine organs, may be affected [8]. Clinical manifestations of overt IO in patients with HH include altered liver enzymes, hepatocellular carcinoma, osteoarthritis, glucose intolerance and diabetes, sexual hormone disorder, cardiac dysfunction and hyperpigmentation, among others [8]. In fact, IO would decrease insulin secretion and increase β-cell apoptosis via iron-induced oxidative stress [9]. Likewise, oxidative stress would be responsible for the liver damage found in IO patients [10,11]. Moreover, IO would be associated with increased atherosclerotic plaque formation and risk of myocardial infarction [12,13]. On the contrary, iron depletion, the most common and effective treatment for IO patients, would decrease low-density lipoprotein (LDL) oxidation and increase high-density lipoprotein cholesterol (HDL-C) levels [13]. Consistently, prior studies carried out by our group showed that HDL from IO patients present both quantitative and qualitative alterations associated with higher risk of cardiovascular disease [14,15].

Absolute telomere length (ATL) is related to aging and cellular damage. As a biomarker, it has been associated with insulin resistance, diabetes and cardiovascular diseases [16,17]. Telomeres are complex DNA–protein structures located at the end of eukaryotic chromosomes which shorten with age in all replicating somatic cells [18] and are associated with different pathological conditions [16,19]. In fact, functional telomeres play a key role in the protection of chromosomes against genome instability [20]. Given that iron might promote oxidative stress [21], which could also be a cause of shorter ATL [22], some studies evaluated the association between ATL and markers of iron metabolism. In fact, in a study of 1174 healthy adults, Tf saturation (TfSat), was associated with shorter ATL [23].

The present study aims to evaluate the relationship betweeniron levelsand ATL in male adults diagnosed with HH.

## Materials and methods

### Subjects and study protocol

Thirty-eight patients with either TfSat > 50% or ferritin concentration > 300 ng/ml were recruited from the Hepatology Division of the University Hospital ‘José de San Martín’ between 2010 and 2012. HH was diagnosed by liver histology compatible with primary IO (iron deposition preferentially within hepatocytes in the periportal region of the hepatic lobule). Among the 38 patients recruited, 13 did not present liver histology compatible with IO and were excluded from the study. Additional inclusion criteria were adult age and male sex. Exclusion criteria were: diabetes, hypothyroidism, cirrhosis, viral hepatitis, HIV infection or cancer. Among the 25 patients with HH included, 8 showed overt IO (TfSat > 50% and ferritin concentration > 300 ng/ml) at the time of the analysis (HH with IO) and the remaining 17 presented TfSat > 50% but ferritin concentration < 300 ng/ml (HH w/o IO). These 17 patients had previously presented IO and had been submitted to a successful phlebotomy to decrease body iron levels. Eight patients were homozygous for the C282Y polymorphism, two were heterozygous for C282Y, one was homozygous for H63D, seven were heterozygous for H63D, one was a compound heterozygote for C282Y/H63D and six were wildtype. Twenty-five age-matched male healthy subjects without clinical or biochemical signs of IO and wildtype for mutations in the *HFE* gene were also included. A single blood sample from each subject was drawn from the antecubital vein after a 12-h overnight fast. Whole blood was used to determine the complete blood count and an aliquot was stored at −20°C to perform the ATL tests. Serum samples were immediately employed for general biochemical determinations**.**

The present study was carried out in accordance with the Declaration of Helsinki and its later amendments or comparable ethical standards. The protocol was approved by the Ethical Committees from University Hospital ‘José de San Martín’ and from the School of Pharmacy and Biochemistry, University of Buenos Aires. All patients signed an informed consent to participate in the study.

### Anthropometric, analytical and genetic procedures

Height and weight were measured with the subject wearing light clothes and without shoes. Body mass index (BMI) was calculated and BMI categories were defined according to the World Health Organization (WHO) adult definition [24]. Complete blood count was carried out in a LH750 autoanalyzer (Beckman Coulter, Fullerton, CA, U.S.A.). Plasma Tf, apolipoprotein (apo) A-I and apo B concentrations were measured by nephelometry (IMMAGE®, Beckman Coulter, Fullerton, CA, U.S.A.). Ferritin concentration was assayed by an electrochemiluminescence assay (VITROS® ECiQ, Ortho-Clinical Diagnostics, Raritan City, NJ, U.S.A.). Serum levels of iron, glucose, triglycerides and total cholesterol as well as the activities of hepatic enzymes were quantified by standardized methods (Roche Diagnostics, Mannheim, Germany) in a COBAS C501 autoanalyzer (Roche Diagnostics, Mannheim, Germany). LDL-C and HDL-C concentrations were determined by selective precipitation methods.

### Measurement of ATL

The ATL measurement was carried out by qPCR as previously described [25], in aStepOne ™ Real-Time PCR System (Applied Biosystems). Genomic DNA was extracted from peripheral blood leukocytes by standard protocols [26]. For each DNA sample, two consecutive reactions were performed: the first one to amplify a fragment of 75 bp of the single copy gene RPLPO (ribosomal protein, large, PO), and the second one for the telomeric sequence. They both were used for standard curve constructions, and allowed the genome/reaction number (S) and the of telomeric sequence/reaction (T) values to be exported from the respective standard curve. The calculation of the T/S ratio resulted in the kbp of telomeric sequence per cell for each individual determination.

### Statistical analyses

The Shapiro–Wilks method was employed to assess data distribution. Normally distributed variables were expressed as mean ± SD and skewed-distributed variables as median (interquartile range [IQR]). Differences were analyzed by ANOVA employing Tukey’s *post-hoc* test; or by Kruskal–Wallis and paired comparison *post-hoc* test according to normal or skewed data distribution, respectively. Significance was defined as *P*<0.05 in the two-tailed tests. For all statistical analyses, the software INFOSTAT (GrupoInfostat, Universidad Nacional de Córdoba, Argentina) was employed.

## Results

### Clinical and metabolic characteristics

**Figure 1:**
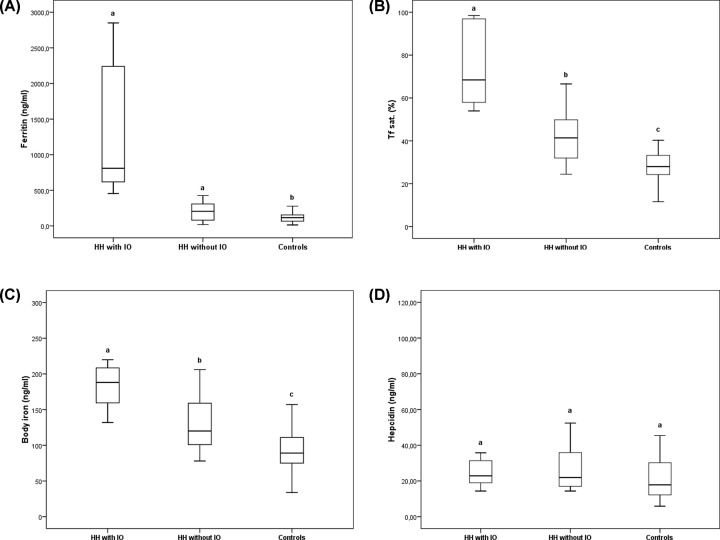
Iron metabolism in IO patients and control subjects Panel A: Ferritin; Panel B: Transferrin Saturation (Transf. Sat.); Panel C: Body Iron; Panel D: Hepcidin. Different letters (a–c) indicate statistically significant differences.

HH patients with overt IO were older (*P*<0.001) and, therefore, statistical analysis was carried out adjusting by age. Furthermore, HH patients with IO also showed higher AST and ALT activities, as well as ferritin concentration than HH patients without IO or control subjects (*P*<0.001) (Table 1). In turn, all these parameters were similar in HH patients without IO and controls. Patients with HH, disregarding the iron status, showed higher BMI and glucose levels than the controls (both *P*<0.01). Finally, TfSat was significantly different in the three groups studied finding the highest values in HH patients with IO and the lowest ones in control subjects (Figure 1). Lipid and lipoprotein profile were similar in all the groups.

**Table 1:** Clinical and metabolic characteristics from IO patients and control subjects

Parameter	HH with IO (*n*=8)	HH w/o IO (*n*=17)	Controls (*n*=25)
Age (y)*	61 (55–67)^1^	44 (34–55)^2^	49 (35–57)^2^
BMI (kg/m^2^)*	28 ± 3^1^	28 ± 2^1^	24 ± 2^2^
Glucose (mg/dl)*	102 (95–114)^1^	95 (90–113)^1^	89 (86–95)^2^
**TG (mg/dl)**	96 (80–133)	113 (77–158)	86 (65–114)
**TC (mg/dl)**	167 ± 32	178 ± 38	186 ± 30
**LDL-C (mg/dl)**	99 ± 20	112 ± 35	119 ± 28
**HDL-C (mg/dl)**	50 ± 13	44 ± 8	48 ± 11
**Apo A–I (mg/dl)**	135 ± 30	127 ± 13	138 ± 31
**Apo B (mg/dl)**	84 ± 20	80 ± 32	94 ± 19
AST (IU/l)*	48 (32–57)^1^	32 (22–45)^2^	19 (18–26)^2^
ALT (IU/l)*	52 (28–72)^1^	28 (21–34)^2^	20 (17–23)^2^
**Albumin (g/dl)**	4.3 ± 0.3	4.4 ± 0.3	4.3 ± 0.1
Hepcidin/ferrtin ratio*	0.03 ± 0.02^1^	0.22 ± 0.21^2^	0.23 ± 0.17^2^
Tf (mg/dl)*	203 ± 38^1^	248 ± 30^2^	249 ± 33^2^

Abbreviations: TC, total cholesterol; TG, triglyceride. * = *P*<0.01. Different superscript numbers over the mean or the median indicate significantly dissimilar groups.

### Iron metabolism parameters

HH patient with IO showed higher levels of ferritin, TfSat (%), and body iron plus lower Tf compared with both HH patients without IO and controls. Additionally, HH patients without IO presented higher TfSat (%) and body iron than control subjects. There were no differences among the groups in hepcidin concentration (Figure 1), but hepcidin/ferritin ratio was significantly lower in HH patients with IO (Table 1).

### ATL in patients with HH

**Figure 2:**
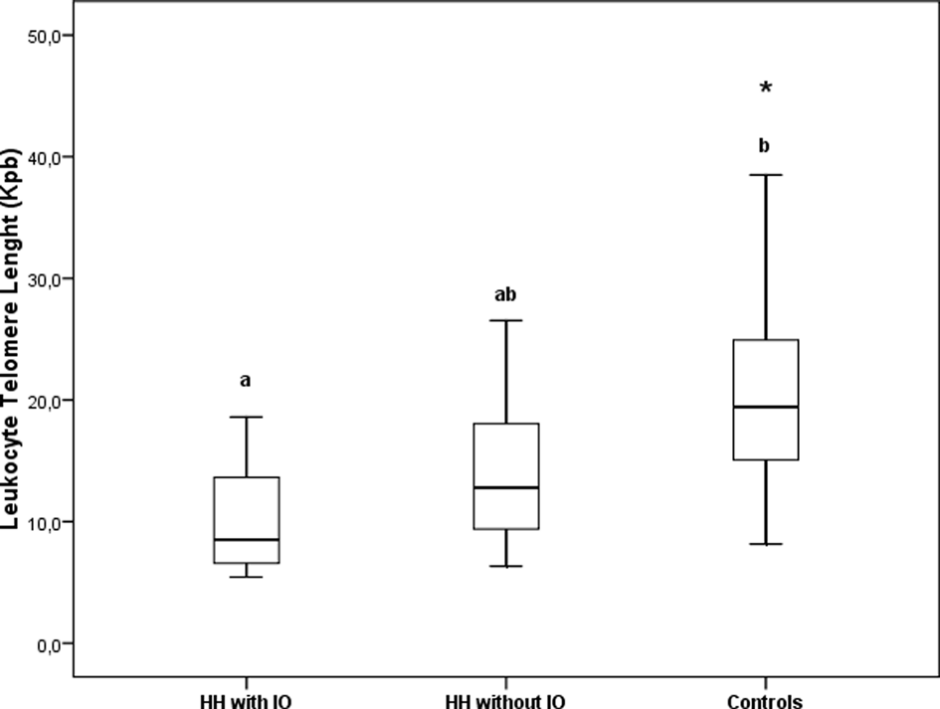
Leukocyte telomere length in healthy controls and IO patients Different letters (a-b) indicate statistically significant differences. *P<0.01.

A linear trend between the groups (*P* for trend<0.01) was observed. ATL were shorter in patients with overt IO than in controls (Figure 2). Differences in ATL remained statistically significant in a model adjusted by age, BMI and glucose levels (*P*<0.05).

ATL was significantly correlated with BMI (r = −0.37; *P*<0.05), glucose (r = −0.26; *P*<0.01), AST (r = −0.36; *P*<0.05), ALT (r = −0.35, *P*<0.05), TfSat (r = −0.26; *P*<0.05), ferritin (r = −0.38; *P*<0.01), and hepcidin (r = −0.28; *P*<0.05). Moreover, no association was detected between HFE genotype/allele distribution and ATL. In multivariate analysis, glucose levels remained the only significant predictor of ATL (r^2^ = 0.32; β = −0.51; *P*<0.01) in a model that also included age, BMI and ferritin.

## Discussion

Telomere shortening can impact tissue homeostasis by impairing cell proliferation and giving rise to genome instability, thus constituting the bases for the development of different pathological conditions [27]. The present study shows that HH patients with overt IO featured significantly shorter ATL while HH patients without IO showed only mild alterations in comparison with healthy controls. The exposition to elevated iron levels over time might be an important factor leading to telomere attrition in HH. The present results highlight the importance of early diagnosis and treatment of IO to preserve ATL.

Excess iron deposition has been shown to be associated with oxidative damage of both circulating biomolecules and cells in a process that accelerates the physiologic changes that naturally occur with aging and leads, among others, to age-related conditions [3–7]. In fact, excess iron accumulation displays multiple toxic effects, which are mostly based on its ability to catalyze the generation of free radicals, resulting in cell death and tissue injury [21]. ROS generation is responsible for the onset of different cellular stress responses such as DNA damage, organelle fragmentation and membrane blebbing. Apart from being directly cytotoxic, iron and free radicals activate inflammatory cells, increasing cytokine production and further generation of ROS [28]. Evidence suggests that free radicals may damage telomere DNA and enhance its shortening [22]. Hence, ATL has been proposed as a marker of cumulative oxidative stress and biological aging [18,22,29].

Apart from the differences observed in the present study in telomere length among the three studied groups, the process of shortening seemed to be associated with the presence of IO. This finding is in agreement with a previous report [30] which showed that iron phenotype and not HFE genotype had a significant effect on telomere length. Patients with IO and, consequently, with the highest values of TfSat and ferritin, showed the shortest telomeres. Furthermore, our study indicates a significant inverse association between TfSat and ferritin levels with telomere length. Similar findings have also been previously reported by Mainou et al. [31], they observed that individuals with high TfSat presented shortened telomere length when compared with those with low TfSat. However, it is important to note that the present study did not include healthy controls and TfSat was the only parameter associated with iron metabolism analyzed in the study. Regarding ferritin levels, Liu et al. [32] observed that telomere length was negatively associated with the presence of high ferritin levels. Nevertheless, in the present study the association between ferritin and ATL was only significant for individuals over 65 years old. Notably, in the present study, ATL was negatively associated with markers of liver damage AST and ALT. This is interesting because the liver is one of the main targets of free radical-induced damage in IO patients [21]. Moreover, in the present study, ATL was inversely correlated with BMI. This finding is consistent with several prior studies carried out by our group which showed increases in BMI and the presence of obesity as major determinants of telomere shortening [33,34].

In addition to oxidative stress, another trait of HH that would contribute to telomere shortening, is inflammation [35,36]. IO promotes liver inflammation which, in tandem with oxidative stress, could contribute to telomere attrition. In particular, iron deposits could directly alter the phenotype of T cells present in the liver leading to changes in cytokine expression [37]. In this regard, it is noteworthy that, as previously mentioned, ATL was negatively associated with ALT and AST levels. Both ALT and AST are considered markers of liver inflammation [38] and would suggest a link between the later and telomere shortening in IO patients.

Remarkably, in the multivariate analysis, glucose levels were the only independent predictor of ATL. This is an interesting finding because it raises the possibility that telomere shortening in IO patients could be the result of alterations in carbohydrate metabolism commonly observed in these patients. Telomere shortening is known to be a feature of diseases that primarily result from alterations in glucose homeostasis such as insulin resistance and diabetes [17]. Nevertheless, to our knowledge, this would be the first study to suggest that impairment in carbohydrate metabolism could be the main factor responsible for telomere shortening in pathologies with high glucose as a secondary trait as is the case with IO. Thus, on these bases, patients with chronic conditions that are frequently associated with hyperglycemia would be also prone to undergo a process of telomere shortening.

As far as we know, our study is the first to show that IO patients who underwent successful treatment aimed to reduce body iron levels and, therefore, systemic oxidative stress [39,40], present ATL values statistically similar to those of healthy controls. This is consistent with previous findings, both *in vivo* and *in vitro*, that indicate a positive effect of antioxidant therapy on telomere length [41,42]. Previous studies showed that the addition of N-acetylcysteine to both human lymphocytes exposed to irradiation and human astrocytoma cells infected with the human immunodeficiency virus decreases the rate of telomere attrition [43,44]. Similarly, serum drawn from subjects under a low-saturated fat diet showed the slowest rate of telomere shortening when added to the culture medium of human umbilical vein endothelial cells [45]. Furthermore, several studies reported an increase in telomere length after antioxidant supplementation in animal models [46,47]. Moreover, different double-blind, randomized placebo-controlled studies suggested that supplementation with ω-3 fatty acids could slow telomere attrition in both overweight patients and older-age individuals [48,49]. Similarly, Zhu et al. [50] observed a significant decrease in telomere attrition in overweight individuals after 4 months of vitamin D supplementation.

The fact that iron depletion could decrease telomere attrition is noteworthy given that the latter has been associated with shorter lifespan as well as a wide variety of age-related diseases and conditions, including cardiovascular disease, diabetes, insulin resistance and hypertension [30,51–54]. Regarding cardiovascular disease, telomere length has been proposed as a predictor of coronary heart disease events [55,56] and cardiovascular disease-related mortality [57,58]. In fact, telomere length would be associated with subclinical atherosclerosis markers such as carotid artery intima media thickness [59,60] and carotid artery calcification [51,61].

In summary, we observed an association between ATL and IO overt condition. HH patients with overt IO featured significantly shorter ATL, while HH patients without IO showed similar ATL when compared with healthy controls.

## Conclusion

In conclusion, overt IO was associated with shorter ATL in patients with HH. Screening for IO is encouraged to prevent iron-associated cellular damage and early telomere attrition. ATL could provide prognostic information for IO-related complications in patients with HH.

## Abbreviations

ALT: alanine aminotransferase
Apo: apolipoprotein
AST: aspartate aminotransferase
ATL: absolute telomere length
BMI: body mass index
HDL-C: high-density lipoprotein cholesterol
HFE: homeostatic iron regulator
HH: hereditary hemochromatosis
IO: iron overload
LDL: low-density lipoprotein
Tf: transferrin
TfR: Tf receptor
TfSat: Tf saturation
